# Shock Synthesis of Five-component Icosahedral Quasicrystals

**DOI:** 10.1038/s41598-017-15771-1

**Published:** 2017-11-15

**Authors:** Julius Oppenheim, Chi Ma, Jinping Hu, Luca Bindi, Paul J. Steinhardt, Paul D. Asimow

**Affiliations:** 1 Division of Geological and Planetary Sciences, California Institute of Technology, 1200 E. California Blvd. M/C170-25, Pasadena, CA 91125 USA; 2 Dipartimento di Scienze della Terra, Università degli Studi di Firenze, Via La Pira 4, I-50121 Firenze, Italy; 3 CNR-Istituto di Geoscienze e Georisorse, Sezione di Firenze, Via La Pira 4, I-50121 Firenze, Italy; 4 Department of Physics, Princeton University, Jadwin Hall, Princeton, NJ 08544 USA; 5 Princeton Center for Theoretical Science, Princeton University, Princeton, NJ 08544 USA

## Abstract

Five-component icosahedral quasicrystals with compositions in the range Al_68–73_Fe_11–16_Cu_10–12_Cr_1–4_Ni_1–2_ were recently recovered after shocking metallic CuAl_5_ and (Mg_0.75_Fe_0.25_)_2_SiO_4_ olivine in a stainless steel 304 chamber, intended to replicate a natural shock that affected the Khatyrka meteorite. The iron in those quasicrystals might have originated either from reduction of Fe^2+^ in olivine or from the stainless steel chamber. In this study, we clarify the shock synthesis mechanism of icosahedral quasicrystals through two new shock recovery experiments. When CuAl_5_ and Fe^2+^-bearing olivine were isolated in a Ta capsule, no quasicrystals were found. However, with only metallic starting materials, numerous micron-sized five-component icosahedral quasicrystals, average composition Al_72_Cu_12_Fe_12_Cr_3_Ni_1_, were found at the interface between CuAl_5_ and stainless steel, demonstrating nucleation of quasicrystals under shock without any redox reaction. We present detailed characterization of recovered quasicrystals and discuss possible mechanisms for generating sufficiently high temperatures to reach melting with relatively weak shocks. We discuss the implications of our five-component quasicrystal for the stability of quasicrystals, which have previously only been considered in alloy systems with four or fewer components. Even small amounts of additional metals expand the stability range of the icosahedral phase and facilitate routine syntheses without extraordinary precision in preparation of starting mixtures.

## Introduction

Quasicrystals are a novel class of solid materials with quasiperiodic translational symmetry and rotational symmetries forbidden in periodic crystals^1–3^. Three types of natural quasicrystal have been discovered, all found in (and, thus far, *only* in) the Khatyrka meteorite^4–7^. The first natural quasicrystal, with composition Al_63_Cu_24_Fe_13_ (all formulas herein are on an atomic percent basis) was named icosahedrite for its icosahedral symmetry, featuring six five-fold axes of rotational symmetry^4^. The second, with composition Al_71_Ni_24_Fe_5_, was named decagonite for its single 10-fold rotational symmetry axis^6^. Finally, a second icosahedral phase, Al_62_Cu_31_Fe_7_, outside the known stability range of the quasicrystalline phase in the Al-Cu-Fe system and provisionally called i-phase II, has also been found in Khatyrka meteorite samples^7^. The occurrence of natural quasicrystals in a meteorite demonstrating evidence of a high-pressure shock and rapid post-shock cooling^8^ suggests that the passage of a shock wave facilitates nucleation and growth of quasicrystals and perhaps relaxes the constraints on precise ratios of starting materials observed in static synthesis methods.

The observed association between natural quasicrystals and natural shock events motivated Asimow *et al*.^9^ to attempt a shock recovery experiment on starting materials resembling those found in Khatyrka. Lacking initial knowledge of what materials would be most suitable, this experiment featured a stack of potential starting materials, all in a stainless steel (SS304, Fe_71_Cr_18_Ni_8_Mn_2_Si_1_ with traces of C, S, and P) container. From front to back along the shock transit direction, the chamber contained olivine (Mg_0.75_Fe_0.25_)_2_SiO_4_, CuAl_5_ alloy, a piece of the Canyon Diablo meteorite (dominantly FeNi metal with possibly some troilite), and a bronze with composition Al_40_Cu_40_Fe_10_Ni_10_. This experiment successfully yielded grains up to 10 μm in size of a five-component icosahedral quasicrystal (i-QC) with compositional range Al_68–73_Fe_11–16_Cu_10–12_Cr_1–4_Ni_1–2_, located at interfaces between stainless steel, olivine, and CuAl_5_. Often the quasicrystals surrounded rounded cores of olivine (Asimow *et al*., 2016), suggesting a possible role for silicate minerals or melt as nucleation sites. Because both Fe^2+^-bearing olivine and metallic Fe-rich steel were found in contact with the quasicrystal, a key aspect of the synthesis mechanism in this experiment remained unclear. The iron in the quasicrystals might have originated either as metallic Fe in the stainless steel or as ferrous iron in the olivine. If the iron originated from the olivine, then there would have to be an Fe reduction process, compensated by oxidation of some other component (e.g., Al^0^ → Al^3+^ from metal to a newly formed generation of spinel and/or corundum) and possibly associated with a thermite-type exothermic reaction^10^. If the iron originated from the stainless steel, then there would be no redox reaction, but the synthesis would still have likely required melting or partial melting driven by some other heat source.

Here we report and characterize two new shock recovery experiments that were designed to resolve the ambiguity in the Fe-source and reaction mechanism associated with shock synthesis of icosahedral quasicrystals. The first experiment, shot S1233, juxtaposed the same olivine composition and CuAl_5_ alloy as before, but encased in a tantalum liner within the SS304 outer recovery chamber, in order to eliminate the metallic iron source. The second experiment, shot S1234, contained only CuAl_5_ alloy placed directly in contact with SS304, eliminating both ferrous iron and silicate mineral or melt nucleation sites. In the following sections, we describe the detailed experimental and analytical procedures; the failure of S1233 and the success of S1234 to form icosahedral quasicrystals; and the characterization of the S1234 products by scanning electron microscopy (SEM) including energy dispersive X-ray spectroscopy (EDS) and electron backscatter diffraction (EBSD), electron probe micro-analysis (EPMA), and transmission electron microscopy (TEM) including EDS and selected-area electron diffraction (SAED). We then discuss the heating mechanisms that might have brought the shocked materials in S1234 to their melting point without thermite reactions, and the consequences of ready recovery of five-component icosahedral quasicrystals for theories of stability previously applied to simpler alloy systems.

## Results

### S1233, SEM analysis

**Figure 1:**
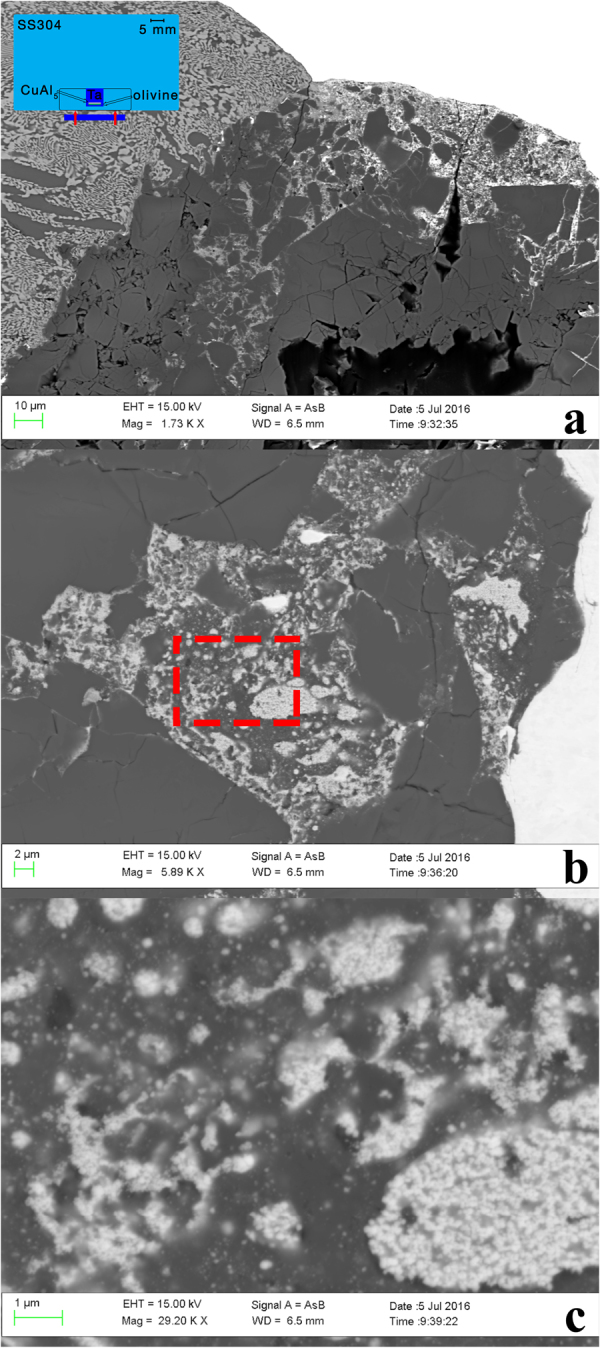
Backscattered electron images of shot S1233. The initial shock propagated from bottom to top in this image. (**a**) Low magnification view of the sample close to rear chamber wall. Dark gray fractured grains are olivine. Black areas are voids from plucking during polish. White areas are Ta. The finely-mixed hypoeutectic CuAl_2_ and Al grain structure of the CuAl_5_ alloy is visible at upper left. Injection of Ta and CuAl_5_ material into fractures in olivine is evident. The inset is a schematic (to scale; 5 mm scale bar shown) of the experimental assembly. (**b**) Higher magnification view of mixed region along the right side-wall of exposed sample chamber showing amorphous oxide material between rounded blebs of Ta. (**c**) Further magnification (area of dashed red box in (**b**)) shows fine dispersion of Ta in amorphous Mg-Fe-Al-O phase.

No quasicrystals were found in this shock recovery experiment. Intimate mixing of Ta, CuAl_5_, and olivine is observed near the radial edges of the sample chamber. The composition of the starting olivine was (Mg_0.75_Fe_0.25_)_2_SiO_4_ (i.e., Fo_75_) but SEM analyses in the region of mixing show that the recovered olivine is Fo_80–90_. The change indicates that some fraction of the Fe^2+^ in the olivine did undergo reduction, a necessary but not sufficient condition for formation of Al-Cu-Fe quasicrystals in this experiment. At one location on the studied polished surface cut through the recovered sample, there is further evidence of the redox reaction that occurred. In the site depicted in Fig. 1, there is very fine-grained material that mainly consists of Mg, Fe, Al, and O, qualitatively consistent with being (Mg,Fe)Al_2_O_4_ spinel or an amorphous equivalent. As all the Al in the starting materials is derived from the metallic state in CuAl_5_ alloy, there appears to have been coupled oxidation of Al and reduction of Fe. In other regions, despite mixing between the olivine and CuAl_5_, no perceptible reaction occurred, possibly because the grainsize is smaller than one micron and no discernible diffraction could be observed in the EBSD patterns, and possibly because sufficient temperature to initiate the thermite reaction was not achieved.

### S1234, SEM and EPMA analysis

**Figure 2:**
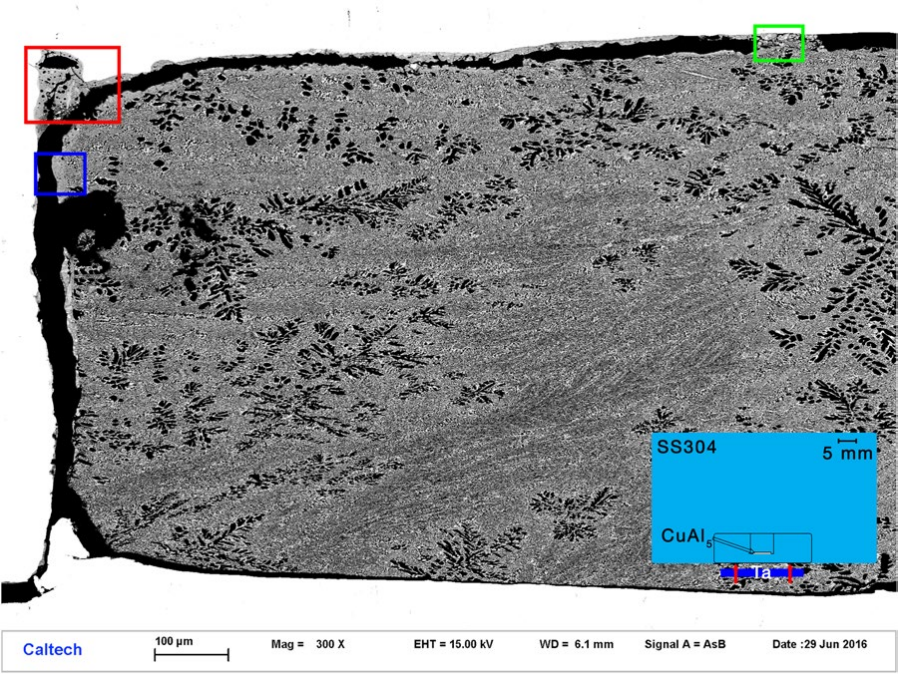
Backscattered electron survey image of recovered sample S1234. The white regions around the margins are SS304. The dendritic region occupying the sample chamber is CuAl_5_ alloy. Black regions are voids where material was lost during sample preparation. ROI 1, shown by the red box, is enlarged in Figs 3b and 4. ROI 2, shown by the green box, is enlarged in Figs 3a and 5. The area along the side-wall highlighted by the blue box is enlarged in Fig. 3c. The inset shows a schematic (to scale; 5 mm scale bar shown) of the entire recovery chamber and flyer plate.

**Figure 3:**
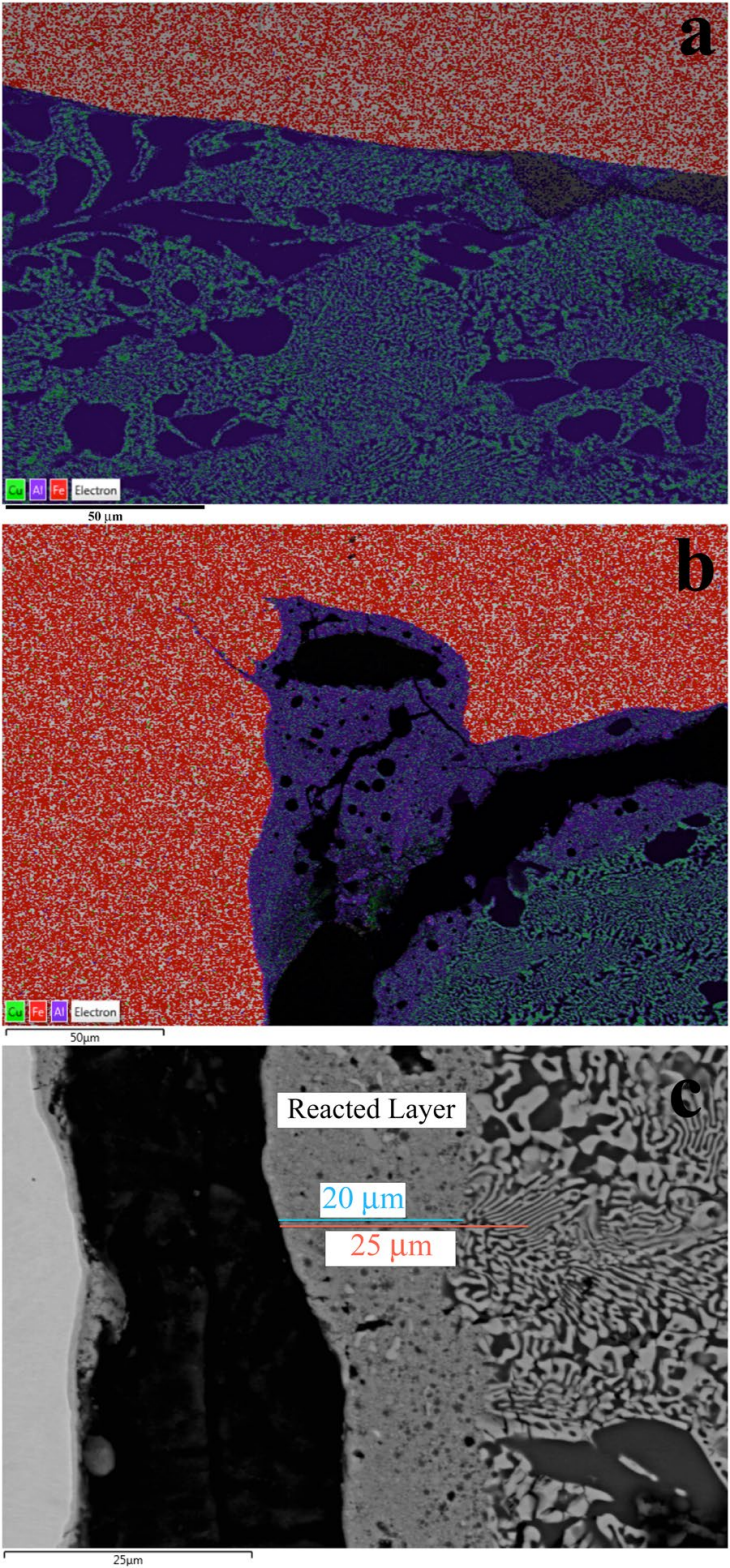
Experiment S1234. (**a**) X-ray intensity map overlain on backscattered electron image, showing a segment of the rear wall of the capsule (green box in Fig. 2). The color scheme is shown at lower-left: Cu is green, Al is purple, Fe is red. Cu-Al alloys are blue-green. Most of the interface is sharp, without evidence of mixing or reaction, except for two patches at right, enlarged in Fig. 5 (ROI 2) that indicate the presence of Fe in a portion of what was originally the CuAl_5_ layer. (**b**) A similar map of the corner formed by the rear wall and the lateral edge of the sample chamber (red box in Fig. 2, also shown in Fig. 4). There is a larger region of mixing and reaction (blue). (**c**) Back-scattered electron image of a segment of the left lateral wall of the sample chamber (blue box in Fig. 2). The bright area at far left is the SS304 chamber wall. The black area is missing material, lost during sample preparation. The eutectoid region at far right is the typical texture of the CuAl_5_ alloy. The 20 μm wide band down the center of the image is a fine-grained reacted zone containing Cu, Al, and Fe.

The general appearance of the recovered capsule is shown in a survey backscattered electron image in Fig. 2. X-ray mapping of S1234 shows only metallic elements; no oxygen is detected, as expected from the all-metallic starting materials. There is thus no evidence of any oxidation or reduction processes. The areas of greatest interest in S1234 are the rear and lateral boundaries between the CuAl_5_ layer and the SS304 capsule. Along most of the rear boundary, the contact remains sharp, with no evidence of mixing or reaction (Fig. 3a), however along parts of this boundary, particularly at the corners where the rear and radial edges meet, mixing is evident in an X-ray intensity map, showing the formation of pockets containing Cu, Al and Fe in significant amounts (Fig. 3b). There is also a well-defined mixed layer along the lateral boundary of the capsule, with a nearly constant thickness of 20 μm (Fig. 3c). Mostly these mixed regions are too fine-grained, <1 μm, for analysis of their crystal or quasicrystal structure at SEM resolution.

**Figure 4:**
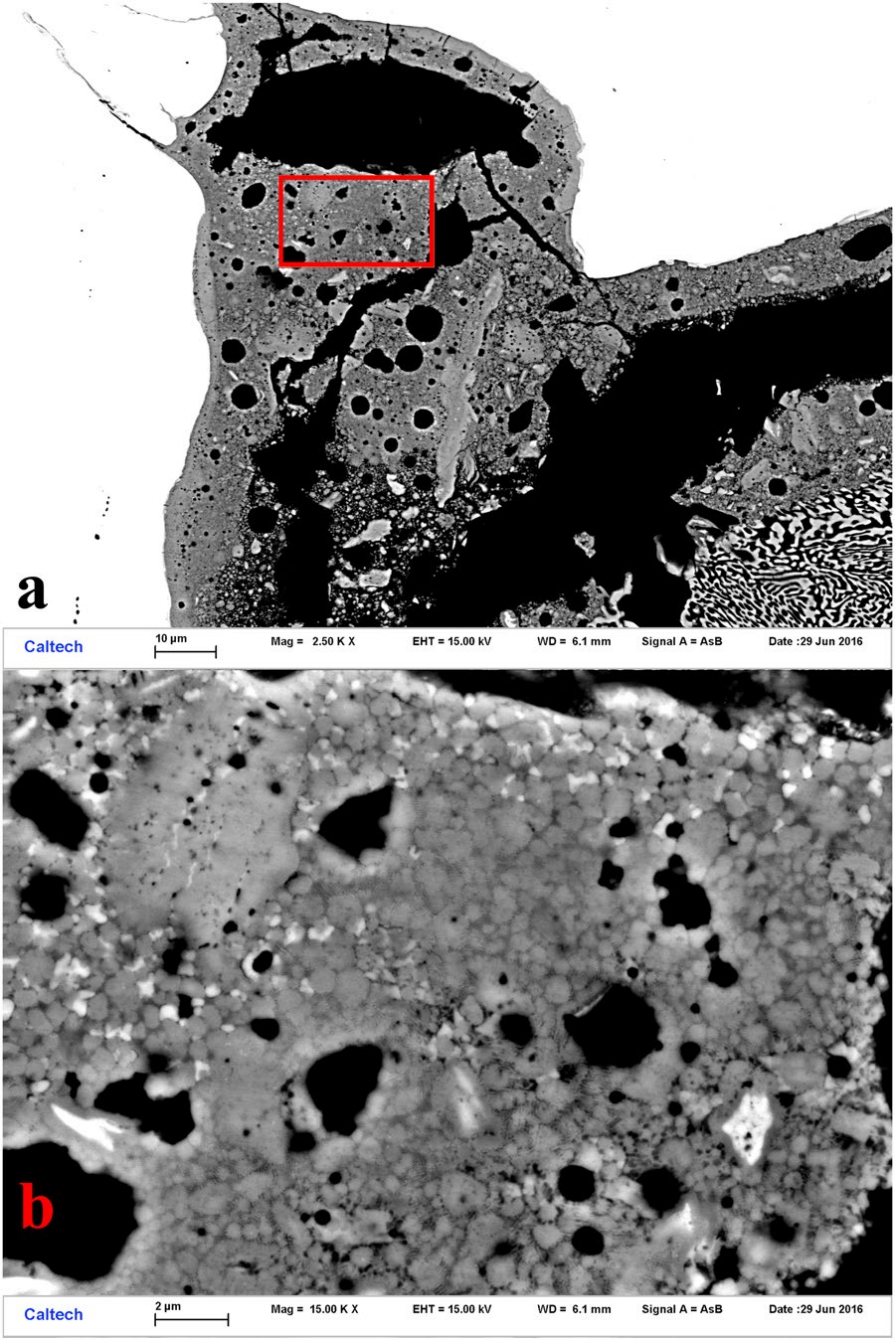
Back-scattered electron images of Region of Interest 1 in shot S1234, from the upper left corner of Fig. 2 (also shown in Fig. 3b). (**a**) Moderate magnification, with SS304 capsule, CuAl_5_, and mixed region evident. Grains approaching 1 μm in size of various contrast levels are visible in the mixed region. (**b**) High magnification image of area highlighted by red box in part (**a**), with voids, scattered Fe-rich grains, and abundant, equant medium-contrast quasicrystal grains.

**Figure 5:**
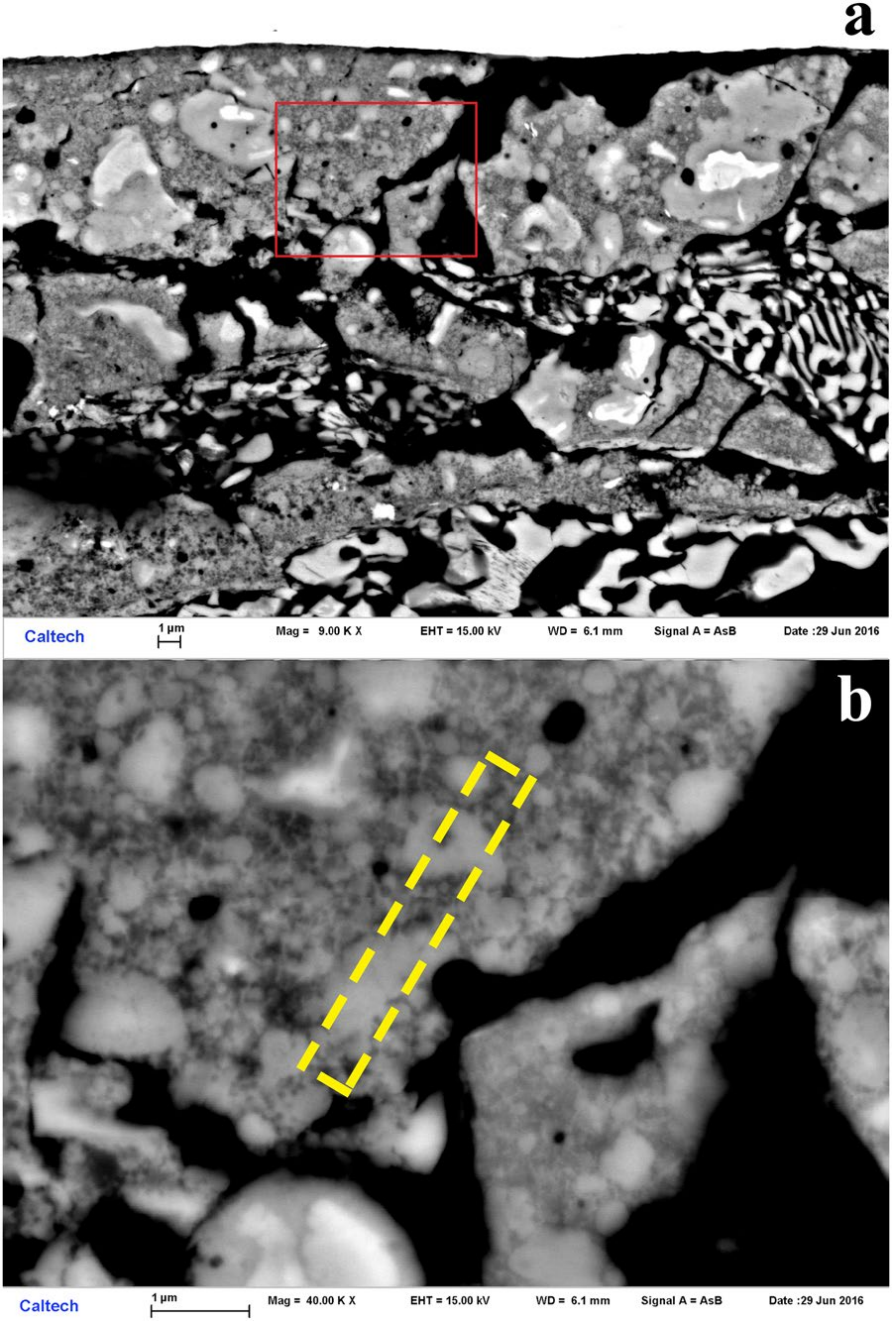
Back-scattered electron images of Region of Interest 2 in sample S1234, along the back wall (also shown in Fig. 3a). (**a**) Moderate magnification image shows relatively coarse grain structure with domains reaching several μm in size. (**b**) High-magnification image of the area shown by the red box in (**a**), with medium grey quasicrystals of order 1 μm in size indicated. The area removed by Focused Ion Beam milling for Transmission Electron Microscopy is indicated by the dashed yellow rectangle.

**Figure 6:**
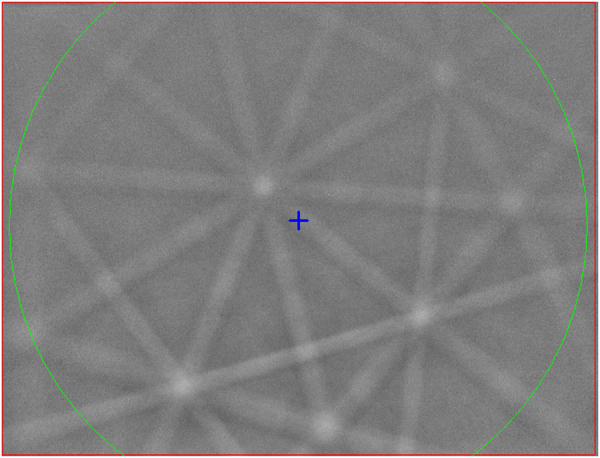
Kikuchi pattern obtained by electron backscatter diffraction from an icosahedral quasicrystal in S1234, displaying a five-fold rotation axis, three two-fold rotation axes, and a three-fold rotation axis.

Close examination of the mixed patches along the rear wall of the capsule and at the corner reveals some coarser-grained areas where grains can be resolved that are amenable to EBSD analysis. The area shown in Fig. 3b and progressively enlarged in Fig. 4 we will designate region of interest (ROI) 1. The area shown in Fig. 3a and progressively enlarged in Fig. 5 we will designate ROI 2. In both these ROIs we observe a significant density of ~1 μm grains of moderate backscatter contrast surrounded by a fine dendritic Al-rich mat and occasional Fe-rich grains (Figs 4c and 5b). These regions were analyzed by EBSD. Some moderate backscatter grains were too small to yield good EBSD patterns, but many of the grains with similar backscatter contrast display Kikuchi patterns with obvious pentagonal symmetry (Fig. 6), forbidden for periodic crystals and strongly suggestive of icosahedral quasicrystalline diffraction. We further studied some of the grains by TEM analysis to refine the structure determination; see below. The Fe-rich grains (>20 atomic %) yield cubic crystal structures in EBSD analysis, but pattern quality is not good enough to distinguish the particular space group.

The grains in each ROI that yielded clear pentagonal zone axes in their Kikuchi patterns were selected for EPMA analysis. Acceptable analytical totals were obtained on 7 grains in ROI 1 and 3 grains in ROI 2. Averages and standard deviations of these compositional analyses are given in Table 1. The spots within each ROI are consistent with being homogeneous populations, however the quasicrystals in the two ROIs are significantly different from one another. The global average of all the EPMA analyses on an atomic basis is Al_72_Cu_12_Fe_12_Cr_3_Ni_1_. Fe, Cr, and Ni are incorporated into the quasicrystal in approximately the same proportions as in the SS304 starting material, but the Al/Cu ratio of the quasicrystal is notably higher than the ratio in the CuAl_5_ starting material. In fact, CuAl_5_ consists of a fine, dendritic mixture of 3 parts Al and 1 part CuAl_2_ (on a per-atom basis). One can successfully describe the composition of the synthesized quasicrystal by bulk incorporation of the three starting phases, i.e. 48 Al atoms from Al, 24 Al atoms and 12 Cu atoms from CuAl_2_, and all the Fe, Cr, and Ni atoms from SS304. The reaction leaves excess CuAl_2_, which explains the difference in Al/Cu ratio between the quasicrystal and the starting material. Both sets of compositions and their average have higher Al contents than any known stable Al-Fe-Cu ternary quasicrystals, icosahedrite, or i-phase II. All five elements are confidently detected above their quantification limits. Given the fine grain size of the i-QC, we used a reduced accelerating potential of 12 kV to contain the electron multiple scattering volume (according to Monte Carlo simulations) to a radius <0.8 μm. This is sufficient to ensure that major element X-rays are emitted within the i-QC and accurately quantified at the percent level. However, secondary X-ray fluorescence effects can yield sub-percent levels of apparent concentration of elements present only in surrounding phases. Hence, we confirmed the presence of the minor elements Cr and Ni in the i-QC by TEM analysis, which takes advantage of thin foil transmission geometry to minimize such artifacts.

**Table 1:** Electron Probe analysis of quasicrystals in S1234.

n	ROI 1	ROI 2
	7	3
***Weight percent***		
*Al*	51.8 ± 2.9	57.3 ± 1.1
*Fe*	20.1 ± 1.2	14.4 ± 1.4
*Cu*	19.9 ± 1.3	22.3 ± 1.5
*Cr*	5.2 ± 0.5	3.7 ± 0.3
*Ni*	2.1 ± 0.3	1.5 ± 0.1
*Total*	99.1	99.2
***Normalized atomic percent***		
*Al*	70.3 ± 2.3	75.1 ± 0.5
*Fe*	13.2 ± 1.0	9.1 ± 1.0
*Cu*	11.5 ± 1.0	12.4 ± 0.7
*Cr*	3.7 ± 0.4	2.5 ± 0.2
*Ni*	1.3 ± 0.2	0.89 ± 0.04

(Analyses filtered for analytical totals above 98%; uncertainty reported is one standard deviation).

### S1234, TEM analysis

**Figure 7:**
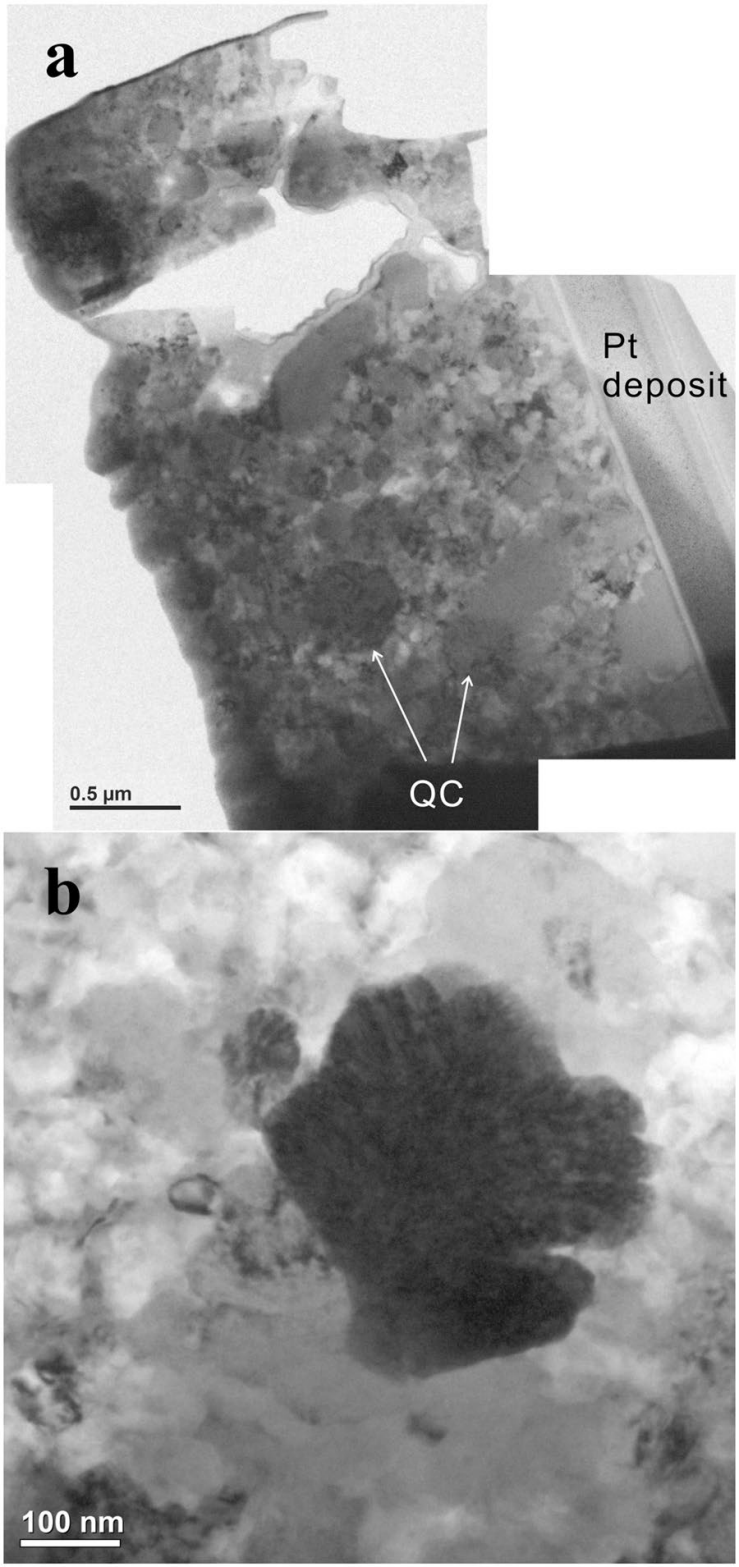
(**a**) TEM Bright-field image of the FIB section. Diffraction contrast results from random orientations of individual icosahedral quasicrystals (i-QC). The arrows annotate large quasicrystal grains. The fine-grained matrix contains i-QCs as well as various crystalline alloys. (**b**) Bright-field image of an icosahedral quasicrystal grain (dark contrast) normal to 5-fold rotation axis.

**Figure 8:**
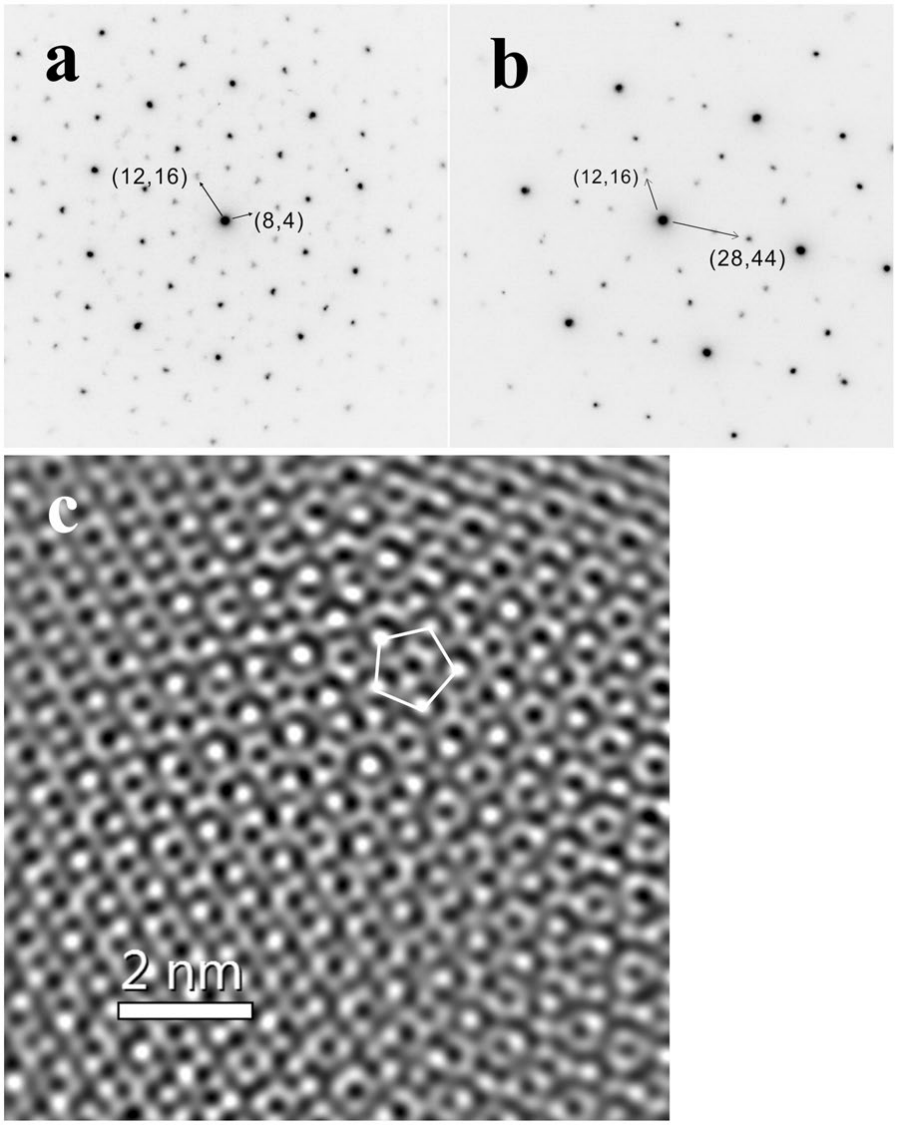
Selected-Area Electron Diffraction patterns along the (**a**) 5-fold and (**b**) 3-fold rotation axes of a quasicrystal grain, obtained by rotating the sample by 36.7°. The Cahn indices of (8,4), (12,16) and (28,44) correspond to 200000, $11\overline{11}11$, and $31\overline{11}11$. The diffracted spots indexed as 200000 are relatively faint. (**c**) Inverse Fourier transform image of frequency-filtered Fourier transform of high resolution TEM image of a quasicrystal from S1234, viewed normal to 5-fold rotation axis. A 5-fold symmetry group is noted with the white pentagon.

A FIB section was obtained from ROI 2, as shown by the dashed white box in Fig. 5b. A bright-field image of the FIB section, Fig. 7a, reveals numerous roughly circular or slightly elongated regions with diffraction contrast resulting from their individual orientations and variable grain sizes from 30 nm to 300 nm. High-magnification bright-field imaging of one such equant grain (Fig. 7b) shows dark contrast due to strong diffraction and some internal contrast. SAED patterns reveal five-fold, three-fold, and two-fold symmetry patterns, as expected for icosahedral symmetry. In one grain that could be rotated to show both a five-fold axis (Fig. 8a) and a three-fold axis (Fig. 8b), the angle between the axes is ~36.7°, consistent with the expectation of 37.38° for ideal icosahedral symmetry. Further evidence that the identified grains are icosahedral quasicrystals can be obtained by examining a high-resolution transmission electron (HRTEM) image. However, FIB milling produces a TEM slice with an amorphous layer from re-deposition of milled materials that interferes with the phase contrast of the quasicrystal. Nevertheless, the Fourier transform (FFT) of the original HRTEM image along the five-fold symmetry axis shows the same diffraction spots as the SAED pattern. After a frequency filter was applied to the transformed image, the inverse Fourier transform image displays a clear quasiperiodic tiling and obvious 5-fold symmetry points (Fig. 8c) characteristic of the icosahedral structure^3^.

Previous authors have noted systematic relations among the composition of icosahedral quasicrystals and their lattice spacings, which can be conveniently expressed by a single lattice parameter in six-dimensional space, *a*_6D_. We attempted to constrain *a*_6D_ for the shock-synthesized quasicrystals using the lengths of the vectors measurable in reciprocal space in our SAED patterns (Fig. 8). Integration of these patterns about the zone centers reveals well-resolved peaks that can be fitted as pure Gaussians. The seven most intense peaks in the integrated patterns (200000, $11\overline{11}11$, $31\overline{11}11$, 420022, $42\overline{22}22$, $53\overline{11}33$ and $62\overline{22}44$), converted from *d-*spacing of each peak to *a*_6D_ by the Cahn indices method^11,12^, yield averaged *a*_6D_ parameters of 12.58 ± 0.21 Å and 12.66 ± 0.22 Å (one sigma uncertainty), for the 5-fold and 3-fold diffraction patterns, respectively. These estimates of *a*_6D_ are consistent with one another and with an extrapolation of the linear correlation between *a*_6D_ and Al content of icosahedral quasicrystals (accurately determined by single-crystal X-ray diffraction at 12.64 ± 0.01 Å for natural icosahedrite with 64 at. % Al and 12.71 ± 0.03 Å for the phase synthesized by Asimow *et al*.^9^ with 71 ± 2 at. % Al), but the precision is too low to add new information to the composition dependence of the six-dimensional cell parameter.

In the transmission electron microscope, it is straightforward to obtain an EDS spectrum from a small grain without significant interference from neighboring material. However, it is not possible to quantitatively measure Cu when the sample is mounted on a Cu TEM grid and internal parts of the microscope are made of Cu. The EDS analysis of the quasicrystals from ROI 2 yields relative proportions of the other metals of Al_72_Fe_12_Cu_X_Cr_4_Ni_1_ (normalized to 72 atoms of Al), which is broadly consistent with the EPMA analyses of the same area. More importantly, the Cr and Ni are unambiguously above detection limit in the quasicrystal and their presence in the EPMA analyses was not the result of contamination from surrounding phases.

## Discussion

### Heating mechanism

According to conventional means of estimating shock and reshock temperatures for non-porous starting materials that experience only simple one-dimensional compression and no other flows^13^, the temperatures that would have been reached during the shock event in S1234 are not expected to exceed 244 °C (see Methods) and post-shock release temperatures would be lower still. These temperatures are well below the melting points, both at the shock pressure and after release to ambient pressure, of all the materials in the experiments. However, there is clear evidence of local melting in parts of the recovered sample chambers, including intimate mixing of discretely layered starting materials, spherical metal blebs, amorphous quench products, and the quasicrystal nucleation and growth process itself. This demands that an additional local heating mechanism operated to increase the temperature. There was no thermite reaction in this all-metal experiment. Here we consider two possible mechanisms that may have affected parts of shot S1234 and attempt to quantify whether either or both could explain the observation that regions of the sample reached the melting points of Al-Cu-Fe alloys.

#### Porosity

Due to imperfect machining and polishing of sample and capsule, there were likely small cavities and voids along the rear interface between the CuAl_5_ disk and the SS304 chamber. When these voids collapse under shock, they allow large amount of local irreversible work that is dissipated as additional heat in the vicinity of the collapsed voids. This can be modeled using a standard continuum porosity model (e.g.,^14^) by approximating the interface region as a thin layer of porous CuAl_5_ sandwiched between the bulk, fully dense CuAl_5_ and the SS304 wall. To raise the predicted single shock temperature of the porous CuAl_5_ layer above 550 °C, the eutectic melting temperature (at ambient pressure) of CuAl_2_-Al mixtures, requires only 0.5% porosity. To further increase the temperature above the melting points of pure Al or Cu at 20 GPa requires 2.5% porosity. A simple interpretation of this result might be that collapse of a series of 0.25 μm pores along the rear capsule wall could easily induce melting of a 10 μm thick layer of CuAl_5_, which is sufficient to explain the observation of mixed patches along the rear chamber wall in S1234.

#### Shear heating as a frictional process

The other place in the capsule that shows evidence of reaction and probable melting is the side-wall or radial outer edge of the sample chamber, which is lined by a 20 μm wide, mostly fine-grained, mixed layer. Because the impedance properties of SS304 and CuAl_5_ are different, the passage of a shockwave propagating parallel to this bi-material interface generates velocity differences across the boundary and hence shear. This is the result both of the different shock velocities in the two materials (such that matching points across the boundary accelerate from rest at different times) and of the different particle velocities (such that they continue to move at different velocities after both have been shocked). Lacking detailed information on the viscosity within the developing mixed layer, it is difficult to quantify a viscous shear-heating model. However, as the flow begins to develop, before any melting begins, we can model it as a dry frictional sliding problem. We model the experiment as a solid cylinder of CuAl_5_ sliding through a hollow cylinder of SS304, and seek to quantify whether the coefficient of friction necessary to melt the materials is plausible.

The rate of heat dissipation into the CuAl_5_ layer, *q*, caused by frictional sliding, per unit area, is

1 $$q=\alpha \mu \,{\rm{\Delta }}P\,{\rm{\Delta }}U,$$

where *α* is the fraction of the heat generation partitioned to the CuAl_5_ side of the boundary (assumed to be 0.5), *μ* is the coefficient of kinetic friction, Δ*P* is the normal stress across the boundary (taken to be equal to the difference between first shock pressures in SS304 and CuAl_5_, 8.8 GPa), and Δ*U* is the velocity difference across the interface (taken to be difference in first-shock particle velocities in the two materials, 184 m s^−1^). The shocked state lasts approximately *t* = 0.7 μ*s*. If the total heat dissipated during this sliding is deposited into a 20 μm thick layer of CuAl_5_, as observed in Fig. 3c and consistent with standard tribology estimates^15^, with (initial) density ~3570 kg m^−3^, the mass per unit area of heated material is *m* = 0.07 kg m^−2^ and the total energy per unit mass dissipated during the sliding event is

2 $${\rm{\Delta }}E=\frac{qt}{m}=8\mu \,{\rm{MJ}}{\rm{.}}$$

The temperature increase, assuming a constant heat capacity *C*_*v*_ = ~700 J kg^−1^ K^−1^ (estimated from the Kopp-Neumann mixing law), is

3 $${\rm{\Delta }}T=\frac{{\rm{\Delta }}E}{{C}_{v}}={10}^{4}\mu \,K.$$

Given bulk shock temperature of 244 °C, reaching a post-shock temperature above the ambient-pressure eutectic point of Al-CuAl_2_ (550 °C) suggests *μ* > 0.03 would be required for melting upon release. On the other hand, reaching the melting points of either pure Al or Cu at 20 GPa (both ~1700 °C) suggests *μ* > 0.14 would be required for melting in the high-pressure shock state. Both estimates are of the right order of magnitude for typical coefficients of friction, which suggests that sliding friction on the side-walls of the chamber is a reasonable means of raising the temperature at these boundaries to melting and explaining the observed mixed layer there.

### Stability of the Quasicrystal

As the composition of the icosahedral quasicrystals found in this study has never been studied before, it is unclear whether it is a stable or metastable phase at ambient conditions. The Al-Cu-Fe abundances alone place it outside the stability field of the three-component (Al-Cu-Fe only) icosahedral phase at ambient pressure. One possibility is that elevated pressure may expand the compositional stability field of the icosahedral phase, just as it has been shown to increase the temperature limits of the icosahedral stability field^16^. Another possibility is that the presence of additional elements might promote stability. Quasicrystal stability is typically examined using one of two empirical methods: Hume-Rothery rules and the cluster line approach. We will attempt to extend each of these methods to five components.

#### Hume-Rothery phase criterion

According to the Hume-Rothery rules, alloys are stabilized by electrochemical, size, and valence electron factors. A Hume-Rothery phase is one that is stabilized by its valence electron concentration, such that the electron density contains a pseudo-gap at the Fermi level due to interference with the Jones zone; this is recognized using the criterion of constant (or nearly constant) values of electrons per atom (*e*/*a*). Given the Fermi energy *E*_*f*_ and the density of states *N*(*E*)^17^,

4 $$(e/a)\equiv {\int }_{0}^{{E}_{f}}N(E)dE.$$

Empirically, (*e/a*) of an alloy can be estimated from the atomic fractions *n*_i_ and the valence *V*_i_ of each element in the alloy using the mixing rule

5 $$(e/a)\approx \sum _{i}{V}_{i}{n}_{i},$$

but the effective valences of transition metals are dependent on alloy composition, which introduces some uncertainty into this analysis. Some commonly used values for valences are shown in Table 2; we will assume Mott & Jones valences in what follows.

**Table 2:** Valence and atomic radii for quasicrystal stability estimates^18^.

	Al	Cu	Ni	Fe	Cr
Mott & Jones Valence	+3	+1	0	−2	−4
Raynor Valence	+3	+1	−0.61	−2.66	−4.66
Atomic radius (pm)	143	128	124	126	128

To develop compositional criteria for quasicrystal stability, we begin with the closure constraint:

6 $$\sum _{i}{n}_{i}=1.$$

Next, icosahedral quasicrystals in the Al-Cu-Fe system have been experimentally shown to be Hume-Rothery phases with (*e/a*) = 1.862 and hence satisfying

7 $$\sum _{i}{V}_{i}{n}_{i}=\mathrm{1.862.}$$

For a ternary system, the solution of equations (6) and (7) is an (*e/a*)-constant line across the ternary, with one degree of freedom. This solution has been valuable in predicting the location of quasicrystals in ternary systems. For a quaternary system, the solution set becomes a plane through the quaternary volume, but this can be reduced using one additional constraint. The Hume-Rothery rules also dictate a limitation on the average atomic size *R*_*a*_^18^, between 137 and 140 pm for icosahedral quasicrystals. Hence, if *R*_*i*_ is the radius of each atom in the alloy (Table 2), the additional constraint is

8 $$137\,{\rm{p}}{\rm{m}} < {R}_{a}=\sum _{i}{R}_{i}{n}_{i} < 140\,{\rm{p}}{\rm{m}},$$

which reduces the solution set for a quaternary system to a narrow band, an approach that again has been used to predict the location of quasicrystals^19^.

**Figure 9:**
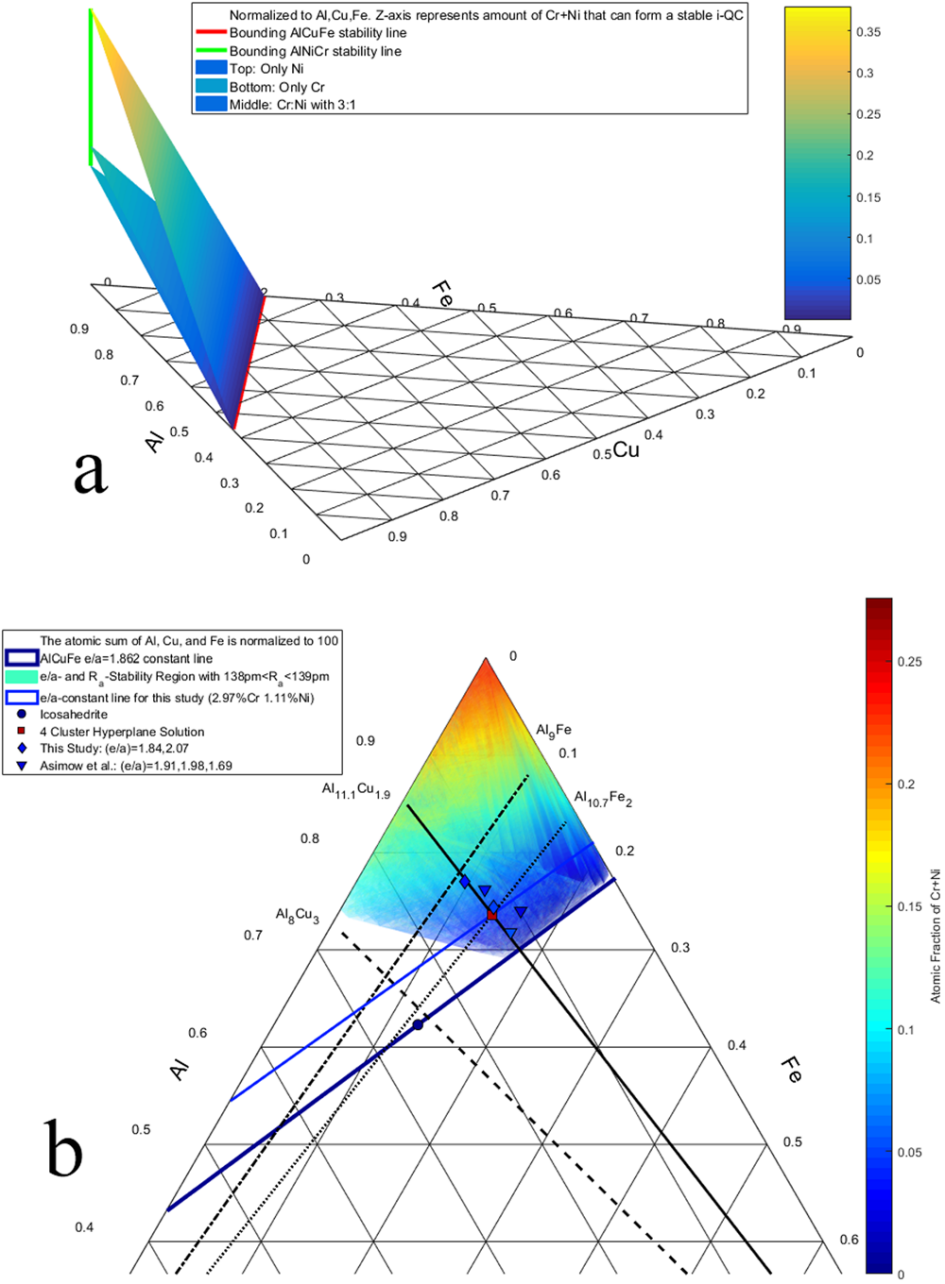
(**a**) Projection of the volume of (*e/a*)-constant stability criteria from five-component Al-Cu-Fe-Cr-Ni space to the ternary Al-Cu-Fe diagram. The planes represent the amount of Ni (alone), Cr (alone), or Ni + Cr in a 3:1 ratio needed in order to have a stable icosahedral quasicrystal. The color shows the atomic fraction of Cr + Ni (Based on ternplot by C. Sandrock). (**b**) The location of experimental and predicted icosahedral quasicrystals in the Al-Cu-Fe-Cr-Ni system, projected onto the Al-Cu-Fe ternary. Features shown include (*e/a*)-constant for ternary Al-Cu-Fe (thick dark blue), (*e/a*)-constant for the atomic fractions of Cr and Ni in this study (thin blue), various cluster lines, Al_63_Cu_24_Fe_13_ icosahedrite (dark blue circle), new compositions from this study (diamonds), new compositions from Asimow *et al*. (2016) (triangles), the 4-cluster hyperplane solution (red square), and the estimated stability region for five-component quasicrystals given by (*e/a*) and size constraints (contoured surface, colors indicate atomic fraction of Cr + Ni).

Assuming that the Al-Cu-Fe-Cr-Ni system has quasicrystal stability criteria analogous to its ternary and quaternary counterparts, the stability region can be bounded using the same constraints. The solution of equations (6) and (7),

9 $${n}_{Fe}+1.4{n}_{Cr}+0.6{n}_{Ni}+0.4{n}_{Cu}=0.2276,$$

is a volume in 5-dimensional space whose projection into the tetrahedral 3-dimensional volume Al-Cu-Fe-(Cr+Ni) (Fig. 9a) is bounded by the surfaces depicting the Al-Cu-Fe-Cr and Al-Cu-Fe-Ni stability fields. Applying the size constraint 138 < *R*_a_ < 139 pm (to avoid negative solutions that occur near 137 or 140 pm) reduces the solution set to a surface, represented in Fig. 9b.

#### Cluster line approach

The stability criteria of quasicrystals have also been considered in ternary systems using a cluster line approach^19^. In this theory, a cluster is a stable binary unit, similar to a unit cell in crystals. The cluster-and-glue model divides the atoms into clusters and glue atoms dispersed between them. On a ternary diagram, a cluster line is drawn between a known binary cluster and the third component. If there are two binary clusters from different pairs of components, the lines will intersect and the intersection composition is usually a stable quasicrystal composition. Some of the binary clusters of Al-Cu-Fe-Cr-Ni are shown in Table 3. In a five-dimensional system, rather than cluster lines, there are cluster hyperplanes, but stability is defined by the intersection of four such hyperplanes, which is still a point in normalized composition space.

**Table 3:** Stable clusters in Al-TM binaries^25^.

Binary	Primary Cluster	Secondary Cluster
Al-Cu	Al_11.1_Cu_1.9_	Al_8_Cu_3_
Al-Fe	Al_10.7_Fe_2_	Al_9_Fe_4_
Al-Cr	Al_11_Cr_2_	Al_12_Cr_1_
Al-Ni	Al_10_Ni_3_	Al_9_Ni_3_

The solution of equation (6) and all four primary cluster hyperplanes is the composition Al_54_Cu_9_Fe_10_Cr_10_Ni_16_ (projected to the Al-Cu-Fe ternary in Fig. 9b). However, given the small abundance of Cr and Ni in the observed quasicrystals, it is probable that the stability is dominated by the Al-Cu and Al-Fe clusters. The intersection of the primary cluster lines for Al-Cu and Al-Fe pairs, the solution of equation (9) and the average size constraint 137.4 pm < *R*_*a*_ < 138.4 pm covers the range Al_67–71_Cu_11–12_Fe_12–13_Cr_0–3_Ni_0–9_.

As shown in Fig. 9b, natural ternary icosahedrite plots along the (*e/a*) = 1.862 line near the intersection of the clusters Al_8_Cu_3_ and Al_10.7_Fe_2_. The Al_8_Cu_3_ cluster is a CN10 octahedral antiprism and Al_10.7_Fe_2_ is an icosahedron. The combination of these two clusters is a Mackay icosahedron. The five-component icosahedral quasicrystals found in this study and those reported in Asimow *et al*.^9^ all plot near the intersection of Al_11.1_Cu_1.9_ and Al_10.7_Fe_2_. Both of these clusters are icosahedra, suggesting that these quasicrystals are composed of Bergman clusters^19^.

At this point, it is unclear whether Hume-Rothery constraints, cluster lines, or other considerations are most significant in the stabilization of five-component icosahedral quasicrystals, although we note that the (*e/a*) values estimated from our electron probe analyses vary over a fairly wide range, from 1.84 to 2.07. The shock synthesis has allowed the discovery of recoverable quasicrystals in a new range of composition space, but further exploration of the bounds of the icosahedral stability field in this area calls for conventional metallurgical synthesis of larger, more uniform crystals that can be better characterized and compared to proposed stability criteria involving (*e/a*), *R*_*a*_, and cluster intersections.

## Conclusion

Five-component icosahedral quasicrystals with the composition range Al_70–75_Cu_9–13_Fe_11–12_Cr_2–4_Ni_1_ were synthesized and recovered by shocking a CuAl_5_ alloy in contact with SS304. This is a simpler set up than that reported by Asimow *et al*.^9^, and demonstrates that oxidation-reduction reactions and oxide or silicate nuclei are not required for efficient shock synthesis of this type of quasicrystal when porosity or shear heating are available to drive melting. Estimation of the six-dimensional lattice parameter *a*_6D_ from TEM SAED remains consistent with the linear correlation with Al contents inferred from previously studied natural and synthetic compositions. We argue that the addition of small amounts of extra components extends the stability range of icosahedral quasicrystals from an extremely narrow and tightly constrained ternary field into a more flexible volume, facilitating synthesis by the rather poorly-controlled shock method. The newly discovered range of recoverable icosahedral phase space confirms the inference about shock synthesis as a means of discovery of new quasicrystal compositions^9^, and also suggests a direction for further testing of the competing empirical hypotheses about the bounds on quasicrystal stability. Although we have not explored the upper or lower bounds on shock pressure needed for i-QC synthesis and cannot easily place any new bounds on the shock conditions experienced by the Khatyrka meteorite, we can confirm that temperature sufficient to melt and mix the starting materials is required and illustrate that such temperatures occur even in shocks where conventional estimates of shock temperature are far below melting points.

## Methods

### Shock Recovery Experiment

In each experiment, discs of starting material 5 mm in diameter and 1 mm total thickness were loaded into a custom-machined cavity 5 mm below the impact surface of a SS304 recovery chamber. In S1233, a 1 mm deep well in a Ta inner screw and a Ta lid provided 1 mm of Ta on all sides. In S1234, a 1 mm deep counterbore in the inner SS304 screw of the recovery chamber^20^ formed the sample volume, which was closed by the SS304 driver itself. The target was impacted by a 2 mm thick Ta flyer carried by a 20 mm diameter plastic sabot. We sought to replicate the conditions of the experiment described in Asimow *et al*.^9^, which had a projectile velocity measured by dual laser interrupts of 988 ± 4 m s^−1^. Therefore, each experiment used a mass of gunpowder (*C*) calculated from the measured weight of the projectile (*M*) using the same *C*/*M* ratio (0.53) as before. Unfortunately, during both experiments reported here, the laser interrupts were not correctly recorded and no velocity was measured. We therefore estimated the uncertainty on projectile velocity from historical data on the same gun and the Le Duc theory^21^, which suggests that muzzle velocity should scale with the square root of *C*/*M*. We regressed the measured velocities in 190 experiments using the Caltech 20-mm bore propellant gun against (*C*/*M*)^1/2^, and found the standard error and prediction interval of the velocity estimate at *C*/*M* = 0.53 both to be ± 162 m s^−1^. We therefore assign projectile velocity of 988 ± 162 m s^−1^ m/s to both S1233 and S1234. Given this velocity estimate and the known Hugoniots of Ta, SS304, and olivine and an estimate of the CuAl_5_ Hugoniot from mixture theory and the shock properties of Cu and Al^22–24^, we estimated the shock states for single shocks with analytical propagation of the uncertainties (Tables 4 and 5). However, given the thickness of the flyer and the duration of supported shock, there were reverberations within the low-impedance sample capsules. Tables 4 and 5 therefore show the estimated pressure and temperature of both the first shock state and the peak or maximum state reached after multiple shocks in each layer. In shot 1234, for example, there was time (~0.67 μs) for at least four reverberations, which would have raised the pressure, in the CuAl_5_ layer, stepwise, to a pressure very close to the capsule pressure of 25.4 ± 4.8 GPa and an estimated peak temperature ≤244 °C.

**Table 4:** First and peak shock states in experiment S1233.

	SS304	Ta	OLIVINE	CuAl_5_	Ta
P_FIRST_ (GPa)	25.4 ± 4.8	31.2 ± 6.0	16.9 ± 3.2	15.4 ± 2.9	24.5 ± 4.7
T_FIRST_ (°C)	134	144	80	158	129
P_PEAK_ (GPa)	31.2 ± 6.0	31.2 ± 6.0	25.4 ± 4.8	24.5 ± 4.7	24.5 ± 4.7
T_PEAK_ (°C)	169	144	126	300	129

Estimates from successive impedance matches with analytical error propagation dominated by the uncertainty in projectile velocity. A 2 mm thick Ta flier launched at 988 ± 162 m s^−1^ impacted, in sequence, a 5 mm thick SS304 driver, a 1 mm Ta capsule lid, an 0.5 mm thick olivine crystal, an 0.5 mm thick CuAl_5_ alloy layer, and a 2 cm thick Ta inner screw.

**Table 5:** First shock states in experiment S1234.

	SS304	CuAl_5_	SS304
P_FIRST_ (GPa)	25.4 ± 4.8	16.5 ± 3.1	22.8 ± 4.3
T_FIRST_ (°C)	134	146	120
P_PEAK_ (GPa)	25.4 ± 4.8	25.4 ± 4.8	25.4 ± 4.8
T_PEAK_ (°C)	134	244	134

Estimates from successive impedance matches with analytical error propagation dominated by the uncertainty in projectile velocity. A 2 mm thick Ta flier launched at 988 ± 162 m s^−1^ impacted, in sequence, a 5 mm thick SS304 driver, a 1 mm thick CuAl_5_ alloy layer, and a 2 cm thick SS304 inner screw.

After each shot, the chamber was recovered intact, sawn open along a plane parallel to the shock direction, polished with abrasives down to 0.25 μm and then by 24 hours of vibrational polishing in 30 nm colloidal silica, and examined by scanning electron microscopy, including EDS and EBSD maps and point analyses, by EPMA, and – in the case of S1234 – by extracting a thin section by Focused Ion Beam (FIB) milling for TEM analysis.

### Scanning Electron Microscopy

Scanning electron microscopy was carried out with the California Institute of Technology (Caltech) Geological and Planetary Sciences Division (GPS) analytical facility’s Zeiss 1550VP field-emission SEM. Imaging and EDS analyses used 15 kV accelerating potential and a 60 μm beam aperture. EBSD analyses used 20 kV accelerating potential and a 120-μm beam aperture in high-current mode. EDS spectra were on an Oxford X-max Si-Drift Detector. EBSD patterns were collected with an HKL system. Both were analyzed using the Oxford Instruments AZtec software.

### Electron Probe

Selected areas of the recovered samples were reanalyzed for Al, Cu, Fe, Cr, and Ni on the five-spectrometer JEOL 8200 electron microprobe in Caltech’s GPS analytical facility, using 12 kV accelerating potential, a focused 5 nA beam, 20 s counting times on peak and 10 s on each background position, and pure metal standards. Points with analytical totals less than 98%, which are presumed to result from poor polish or focus on very small grains, were discarded. The Casino Monte Carlo simulation software was used to verify that the activation volume for characteristic X-ray emission with this protocol had a radius of ≤0.8 μm.

### Transmission Electron Microscopy

The FIB and TEM facilities used are in the Kavli Nanoscience Institute at Caltech. A foil was milled and lifted out from the quasicrystal-bearing region of sample S1234 using a FEI Nova 600 Nanolab DualBeam FIB and SEM. After placement on a copper TEM grid, sample thinning was finalized with an 8 kV 19 nA Ga-ion beam. Analytical transmission electron microscopy (ATEM) analysis was performed on a FEI Tecnai TF20 instrument with super-twin objective lenses, operated at 200 kV. The energy dispersive spectroscopy (EDS) data were collected in TEM mode using an EDAX SiLi detector with 10 eV/channel and 51.2 µs processing time, to achieve 500 counts per second signals and 20–50% dead time. The SAED patterns were integrated using Gatan DigitalMicrograph™ to refine the *d*-spacings of the studied quasicrystals.

### Data availability statement

The original analytical data and archived recovered sample products described in the current study are available from the corresponding author upon reasonable request.
